# Nécrolyse épidermique toxique induite par le phénobarbital chez un enfant Rwandais: à propos d'uncas

**DOI:** 10.11604/pamj.2014.17.202.3385

**Published:** 2014-03-14

**Authors:** Célestin Kaputu-Kalala-Malu, Olga Ntumba-Tshitenge, Jean-Paul Misson

**Affiliations:** 1 Service of Child Neurology, Department of Neurology, Kinshasa School of Medicine, University of Kinshasa, Democratic Republic of Congo; 2 Department of pediatrics, Butare University Teaching Hospital, National University of Rwanda, Rwanda; 3 Service of Pediatrics and Child Neurology, CHR Citadelle Hospital and CHU University Hospital, University of Liège, Belgium

**Keywords:** Syndrome de Lyell, phénobarbital, enfant, Afrique, Lyell Syndrome, phenobarbital, child, Africa

## Abstract

Le syndrome de Lyell ou la nécrolyse épidermique toxique (TEN) est une des rares complications majeures du traitement par phénobarbital. Sa prise en charge n'est pas encore codifiée. Il requiert une intervention urgente, lourde et adaptée à chaque patient afin d'en réduire la mortalité. Nous décrivons un cas du syndrome de Lyell survenu une dizaine de jours après initiation du traitement antiépileptique par phénobarbital chez un enfant rwandais de deux ans. La complexité des lésions cutanéomuqueuses et leurs répercussions sur le plan général soulignent l'importance d'une prescription responsable et justifiée des médicaments antiépileptiques.

## Introduction

Le phénobarbital est sans aucun doute la médication antiépileptique la plus ancienne et reste également très souvent prescrit chez le jeune enfant dans cette indication [1]. A ce titre il est repris dans la liste des médicaments essentiels de l'Organisation Mondiale de la Santé de 2013 [2].Malgré son innocuité apparente, il peut induire des effets secondaires allant du plus bénin au plus grave et mortel à l'instar de nécrolyse épidermique toxique (TEN) connue sous le nom de syndrome de Lyell [3]. La TEN est considérée comme une «allergie» médicamenteuse caractérisée par la destruction brutale de la couche superficielle de la peau (épiderme) et des muqueuses (épithélium). On utilise le nom de syndrome de Lyell pour les formes les plus étendues - plus de 30% de surface corporelle - et celui de syndrome de Stevens-Johnson (SJS) pour les formes limitées de nécrolyse épidermique qui peuvent le rester ou progresser vers un syndrome de Lyell [4] Son incidence a été évaluée à environ 1 cas par million d'habitants par an.En Europe, l'incidence conjointe de la TEN et du SJS est de 2 cas par million d'habitants par an [5]. Sa prise en charge, généralement symptomatique et lourde, est indispensable en urgence. Toutefois, le pronostic reste grave, 20 à 25% de mortalité et près de 50% de séquelles, en particulier oculaires, chez les survivants [6]. À notre connaissance, aucun cas de syndrome de Lyell n'a encore été publié en Afrique Centrale.

## Patient et observation

Un enfant de sexe masculin, âgé de 2 ans et demi, pesant 13 kilogrammes, a été admis, via le service d'urgence pédiatrique, au Centre Hospitalier Universitaire de Butare / Rwanda pour une prise en charge des lésions cutanéomuqueuses disséminées évoluant depuis 7 jours. Les antécédents personnels et familiaux n’étaient pas contributifs en dehors de la survenue des crises épileptiques tonicocloniques généralisées pour lesquelles les parents avaient consulté dans un hôpital de district. Une thérapie anticomitiale faite de phénobarbital à la dose de 50 mg par jour (soit 3,8 mg/kg/dose) a été débutée 19 jours plutôt. L’évolution a été marquée, 12 jours plus tard, par l'apparition de la fièvre (dont l'intensité n’était pas documentée) et d'une éruption érythémateuse étendue. Cette phase a été rapidement suivie par l'apparition des lésions cutanéomuqueuses bulleuses disséminées pour lesquelles l'enfant a été référé au Centre Hospitalier Universitaire de Butare pour meilleure prise en charge.

**Figure 1:**
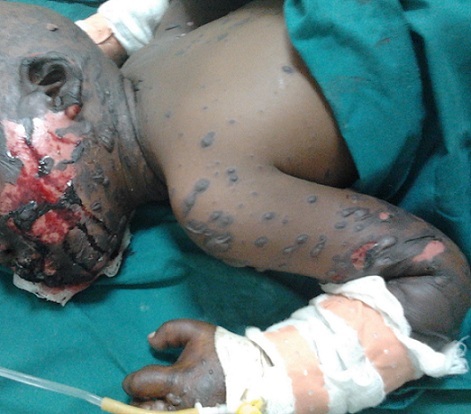
Lésions érosives d'allure nécrotique au niveau de la muqueuse buccale. Lésions cutanées associant bulles flasques et nécrose épidermique laissant le derme à nu au niveau du visage, du cou et des membres supérieurs

**Figure 2:**
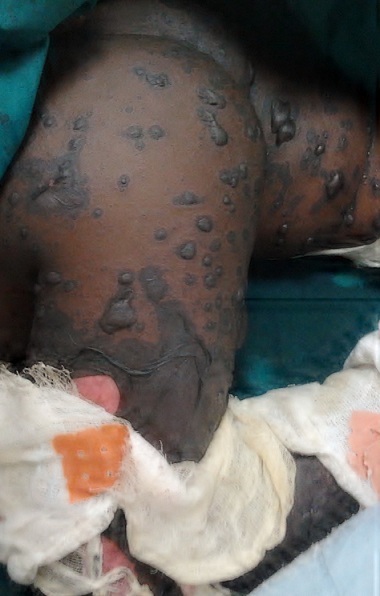
Lésions cutanées associant bulles flasques et nécrose épidermique réalisant un décollement en “linge mouillé” typique au niveau de la ceinture pelvienne et des cuisses

A l'examen physique, l’état général était altéré par la fièvre mesurée à 38,5°C. L'examen dermatologique a révélé des lésions érosives d'allure nécrotique intéressant les muqueuses oculaires et buccales responsables des troubles de vision et d'alimentation. Les lésions cutanées, quant à elles, prédominaient à la tête, au visage, au cou, aux membres supérieurs, à la ceinture pelvienne ainsi qu'aux membres inférieurs. Elles consistaient en des plages des bulles à toit nécrotique d'allure confluant séparées par des plages de nécrose épidermique réalisant un décollement en « linge mouillé » typique avec le signe de Nikolsky (Figure 1, Figure 2 ). La surface corporelle atteinte a été évaluée à 35%. Sur la base des éléments objectifs et anamnestiques, le diagnostic du syndrome de Lyell induit par le phénobarbital a été évoqué. Le score prédictif du pronostic (SCROTEN) était estimé favorable car évalué à 2/7 [7]. La biopsie cutanée pour un examen anatomopathologique n'a pas pu être réalisée pour des raisons logistiques.

### Prise en charge et évolution

D'emblée l'enfant a été admis en soins intensifs dans le but de contrôler de manière rigoureuse les paramètres hémodynamiques et de prévenir les désordres hydroélectrolytiques pouvant survenir à cause de la difficulté d'alimentation et des pertes cutanées. Les lésions de muqueuses buccales ont été prises en charge par un tamponnage quotidien au sérum physiologique et l'application du gel oral Daktarin^R^(Miconazole). La tétracycline ophtalmique suivie d'application des larmes artificielles pour sécheresse oculaire ont constitué l'essentiel des soins oculaires.

La prise en charge des lésions cutanées a consisté en la mise du patient dans une chambre d'isolement pour éviter les surinfections. Les lésions cutanées ont bénéficié de quelques séances de débridements en salle d'opération sous anesthésie générale (Kétamine^R^) et des pansements non adhésifs. L'application de la FLAMAZINE^R^crème (Sulfadiazine d′argent à 1%) et de la crème FUCIDINE^R^ (acide Fusidique à 2%) a été effectuée suivant l’état des lésions et les recommandations des fabricants.

**Figure 3:**
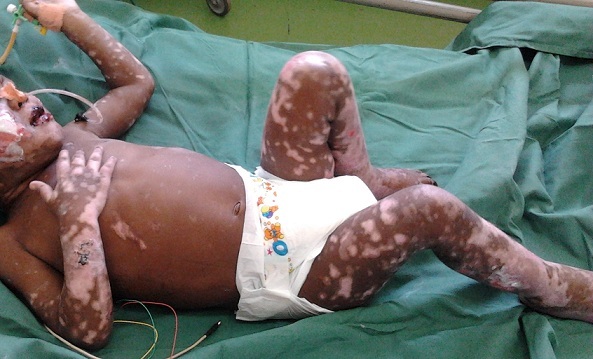
Lésions cutanéomuqueuses en voie de cicatrisation. Au 25ème jour d'admission

Le paracétamol et le tramadol^R^ ont été utilisés dans la gestion de la douleur. L’évolution a été marquée, dès le quatrième jour d'hospitalisation par une hyperthermie variant entre 38 °C et 39,7° C malgré la négativité des investigations paracliniques dont 2 hémocultures, 2 analyses cytobactériologique des urines et 2 gouttes épaisses à la recherche de trophozoïtes. Outre les antibiotiques qui avaient été administrés de manière empiriques (Ampicilline + gentamycine puis céfotaxime et pour finir, amoxilline+ acide clavulanique), cette hyperthermie qui a duré 20 jours a été prise en charge par un enveloppement humide et le paracétamol en alternance avec l'ibuprofène. La cicatrisation avec la régénération de l’épiderme a débuté vers le 14ème jour suivant l'admission du malade laissant les cicatrices hypochromiques (Figure 3, au 25ème jour d'hospitalisation). Les lésions muqueuses ont été les dernières à cicatriser mais le patient à continuer de présenter, avant sa sortie, une sécheresse oculaire malgré l'application des larmes artificielles. Les contrôles des fonctions rénales et hépatiques étaient normaux au début et à la fin du traitement. L'acide valproïque a été proposé en remplacement du phénobarbital.

## Discussion

Des études d'observation cas-témoins ont établi que la cause la plus fréquente des syndromes de Lyell et de Stevens-Johnson, est une « allergie » médicamenteuse, bien établie dans environ 60% des cas, plausible dans environ 30%. Plusieurs médicaments ont été impliqués, au moins une fois, dans la survenue de cette « allergie » mais, moins de dix rendent compte d'environ la moitié des cas en Europe. Il s'agit de la carbamazépine, la lamotrigine, le phénobarbital, la phénytoïne, l'allopurinol, la névirapine, les sulfamides anti-infectieux et les antiinflammatoires non stéroïdiens dérivés de l'oxicam. Parfois, aucun médicament suspect n'est identifié, aucune exposition médicamenteuse n'est retrouvée. Quelques-uns de ces cas d'apparence idiopathique ont été expliqués par des infections, en particulier des pneumopathies atypiques à Mycoplasmapneumoniæ [5, 6]. Le mécanisme conduisant à la mort par apoptose brutale et disséminée des cellules de l’épithélium de la peau et des muqueuses demeure non élucidé. Un phénomène de cytotoxicité lymphocytaire contre des cellules épidermiques reconnues comme étrangères après fixation du médicament responsables de ces réactions sur certaines molécules HLA de classe I est évoqué. Les médiateurs chimiques qui semblent être impliqués dans l'amplification violente et diffuse de l'apoptose ne sont pas encore clairement identifiés [8–10]. Les hôpitaux situés en Afrique Subsaharien ont un triple défi à relever devant cet “effet secondaire” majeur lié à la prise du phénobarbital qui est, sans aucun doute, l'antiépileptique le plus consommé grâce à son prix abordable et compte tenu de l'incidence et de la prévalence élevées de l’épilepsie dans cette partie du monde.

Le premier défi est d'ordre diagnostic. La rareté de ces syndromes et le manque d'un personnel qualifié dans le maniement des antiépileptiques ainsi qu'en la détection rapide des effets secondaires peuvent faire retarder le diagnostic et la mise en branle d'une prise en charge adaptée comme c'est dans le cas que nous venons de décrire.

Le deuxième défi est thérapeutique. La prise en charge des syndromes de Lyell et de Stevens-Johnson n'est pas codifiée, elle requiert parfois une multidisciplinarité (neurologue ou pédiatre, dermatologue, chirurgien esthétique, anesthésiste, stomatologue, dentiste, anatomopathologiste, microbiologiste). Qui n'est pas facile à mettre en place. À part les ressources humaines, les ressources matérielles utiles pour diagnostiquer les complications à temps (troubles électrolytiques, troubles des gaz sanguins) afin de réajuster le traitement en cours ne sont pas toujours disponibles dans les institutions hospitalières les plus fréquentées. C'est dans cet ordre d'idée que nous n'avons pas pu savoir si l'hyperthermie présentée par le patient était seulement dû aux perturbations de la thermorégulation rencontrée dans la TEN [6] ou à une surinfection par un germe nosocomial qui n'a pas pu être isolé.

Enfin, il y a le défi d'ordre prévisionnel. Il sied de rappeler que l’ « éducation à la prise des antiépileptique» fait partie intégrante de la thérapeutique antiépileptique. Ceux qui sont appelés à prescrire les antiépileptiques, notamment le phénobarbital, doivent informer les nouveaux patients sur le risque réel des effets secondaires graves. Ce risque est particulièrement élevé durant les deux premiers mois suivant l'initiation du traitement par phénobarbital [11]. Un accent devrait être mis sur l'arrêt immédiat de la molécule récemment prescrite et la consultation sans délai à la moindre apparition d'une lésion cutanée ou muqueuse non autrement expliquée.

## Conclusion

L'administration des antiépileptiques et particulièrement du phénobarbital doit être faite suivant les indications rigoureuses. La reconnaissance rapide d'une lésion cutanée ou muqueuse non autrement expliquée dans les jours ou semaines suivant l'initiation du phénobarbital doit être suivie de son arrêt immédiat et d'une consultation dans un hôpital mieux équipé afin de réduire la morbidité et la mortalité chez les patients.
